# Heat Shock Proteins in Glioblastoma Biology: Where Do We Stand?

**DOI:** 10.3390/ijms20225794

**Published:** 2019-11-18

**Authors:** Rebeca Piatniczka Iglesia, Camila Felix de Lima Fernandes, Bárbara Paranhos Coelho, Mariana Brandão Prado, Maria Isabel Melo Escobar, Gustavo Henrique Doná Rodrigues Almeida, Marilene Hohmuth Lopes

**Affiliations:** Department of Cell and Developmental Biology, Institute of Biomedical Sciences, University of Sao Paulo, Sao Paulo 05508-000, Brazil; rebecapiglesia@gmail.com (R.P.I.); mila.felixf@gmail.com (C.F.d.L.F.); coelhobarbarap@gmail.com (B.P.C.); maribrandaop@gmail.com (M.B.P.); meloescobarov@gmail.com (M.I.M.E.); henrique.gustavo1436@gmail.com (G.H.D.R.A.)

**Keywords:** heat shock proteins, chaperones, glioblastoma, therapy, stem cells, proteostasis

## Abstract

Heat shock proteins (HSPs) are evolutionary conserved proteins that work as molecular chaperones and perform broad and crucial roles in proteostasis, an important process to preserve the integrity of proteins in different cell types, in health and disease. Their function in cancer is an important aspect to be considered for a better understanding of disease development and progression. Glioblastoma (GBM) is the most frequent and lethal brain cancer, with no effective therapies. In recent years, HSPs have been considered as possible targets for GBM therapy due their importance in different mechanisms that govern GBM malignance. In this review, we address current evidence on the role of several HSPs in the biology of GBMs, and how these molecules have been considered in different treatments in the context of this disease, including their activities in glioblastoma stem-like cells (GSCs), a small subpopulation able to drive GBM growth. Additionally, we highlight recent works that approach other classes of chaperones, such as histone and mitochondrial chaperones, as important molecules for GBM aggressiveness. Herein, we provide new insights into how HSPs and their partners play pivotal roles in GBM biology and may open new therapeutic avenues for GBM based on proteostasis machinery.

## 1. Introduction

Chaperone proteins play a fundamental role in assisting protein synthesis, folding, remodeling, targeting, and inhibition of non-functional and potentially pathogenic protein aggregate formation, being essential players for the maintenance of proteome homeostasis (i.e., proteostasis) [1]. An important and vast group of chaperones is induced by heat shock and is denominated heat shock proteins (HSPs) [2]. Besides their role in proteostasis, HSPs can also act in antigen presentation, hormone receptor assembly, secretion, and cellular trafficking [3]. On one hand, considering the many roles that HSPs are involved in, their malfunction can lead to a disruption in cell proteostasis and death [4,5]. They can also be co-opted by malignant cells to help promote their survival and progression [6]. HSPs present several well described roles in cancer origin, progression, and maintenance, able to orchestrate many different mechanisms with a large number of molecules involved, demonstrating their relevance for tumor biology and, in particular, as potential therapeutic targets.

Growing evidence has pointed out HSPs as key players in dense complexities of hallmarks in cancer biology, such as self-sufficiency in growth signals, evading apoptosis, sustained angiogenesis, and tissue invasion and metastasis, described by Hanahan et al. [7]. One important hallmark of cancer is its self-sufficiency in growth signals [8], which can be mediated by the family of heat shock proteins of 90 kDa (HSP90). HSP90 is a highly abundant and conserved chaperone throughout evolutionary history [9] and is involved in, among other roles, folding membrane receptors and downstream molecules [10]. For example, HSP90 can stabilize HER2, AKT, Raf-1, EGFR (epidermal growth factor receptor), and many other proteins involved in cell proliferation pathways, which could help to drive tumor progression [11,12,13,14].

Heat shock proteins of 70 kDa (HSP70) correspond to a large and very conserved family of chaperones, with orthologs being stress-induced (for example, HSPA1A/HSP70, HSP72, and others) and expressed constitutively (for example HSPA8/HSC70, HSC73 and others) in different cellular compartments, all performing important functions, mainly associated with proteostasis [1,5,15]. HSP70 is capable of interacting with ERα, promoting its expression and leading to an increased growth in MCF-7 breast tumors [16].

Mutated proteins are also an important part of tumor biology, as they can lose or gain new functions that can be the foundation for cancer emergence. In this regard, HSP90 has a preference to bind and stabilize some mutated proteins over their wild-type counterpart, such as p53, a well-known tumor suppressor gene, therefore contributing to mutagenesis [17,18]. Further studies show that HSP70 can also form a complex with mutated p53 [19]. Additionally, HSP90 is able to chaperone proteins formed by chromosomal translocation, such as the oncogene Bcr–Abl synthesized by the Philadelphia chromosome, common in leukemia patients [20].

The ability of tumorigenic cells to circumvent programmed cell death (PCD) is another important hallmark of cancer progression, as not only can it lead to the development of new tumors, but also to chemo-resistance [8]. Our longstanding knowledge on the apoptotic process has been recently exploited to develop new anti-cancer treatments [21]. Interestingly, besides being able to interact with AKT, an upstream activator of caspase-9 [22], HSP90 directly prevents the activation of pro-caspase-9 through the inhibition of cytochrome c [23]. In addition to that, HSP70 can not only bind to APAF-1 (a central hub of apoptosis regulatory network) and inhibit the formation of apoptosomes, but it can also prevent the release of pro-apoptotic factors through the inhibition of Bax, a protein involved in the release of cytochrome c [24]. HSP60 is another HSP with a role in PCD, whereby HSP60 modulates the activation of procaspase-3 and promotes cell survival [25]. Studies have shown that inhibition of HSP70 and HSP27 lead to increased PCD [26,27]. HSP27 can also prevent the activation of pro-caspase-9 in leukemia, and is able to interact with cytochrome c, inhibiting the formation of apoptosomes [28].

A further hallmark of cancer is neoangiogenesis [8]. A few HSPs are known to regulate hypoxia-inducible factor (HIF) 1α, a transcription factor expressed in conditions of low oxygen, such as the core of solid tumors, and to have an important role in initiating hypoxia-induced angiogenesis [29]. Both HSP70 and HSP90 are able to stabilize HIF1α and its downstream factors, such as vascular endothelial growth factor (VEGF) and nitric oxide synthetase, which require HSP90 for induction and stability [30]. In addition, a study showed through co-immunoprecipitation assays that both HSP90 and HSP70 can interact with HIF1α, and that HSP70 is able to bind directly to the oxygen-dependent degradation domain of HIF1α [29]. Interestingly, many studies demonstrate HSP90 as one of the major regulators of HIF1α, emphasizing its important role in the modulation of this pathway [31,32].

Malignant cells are also able to evade the host immune system [8,33], and studies indicate that HSPs might play an important function in this feature. A study using cells from ovarian cancer patients demonstrated that the circulating HSP10 released from the tumor was able to suppress T-cell CD3-zeta expression, important for T-cell activation [34]. Moreover: (1) HSP72 is able to decrease T-cell proliferation and mediate inflammatory responses [35]; (2) as HSPs contribute to antigen presentation via MHC class I and II, they represent a valuable target against tumors [36]. In the last few years, many clinical trials have used HSP–peptide vaccines to treat cancers such as gastric, renal, colon, and pancreatic carcinoma [36]. Indeed, when located on the cell membrane of tumors, HSP70 can be recognized by natural killer (NK) cells and dendritic cells, leading to an enhanced antitumor response [37,38].

The ability to invade and metastasize is another important hallmark of cancer [7]. According to recent experimental studies, HSP90 seems to have an increased expression in aggressive and metastatic tumors, such as melanomas and hepatic tumors [39,40], while also being associated with a higher chance of early recurrence in neuroendocrine tumors [40]. One of the early steps required for invasion and metastasis formation is the epithelial–mesenchymal transition (EMT), a process whereby epithelial cells lose their polarity and are able to assume a mesenchymal phenotype [41]. HSP90 can modulate the expression of fibronectin, as well as interact with matrix metalloproteinase 2 (MMP2) in prostate cancer and fibrosarcoma, respectively [42,43]. Together with HSP90, HSP70 stabilizes and promotes the expression of WASF3, a protein involved in the formation of multiprotein complexes modulating cell shape and migration and ultimately leading to increased metastasis in breast cancer [44]. Finally, HSP-organizing protein (HOP), a co-chaperone of HSP90/HSP70, is overexpressed in pancreatic tumor cells, and a decrease in HOP levels leads to a concomitant decrease in MMP2 expression, thus modulating invasion [45]. 

**Figure 1:**
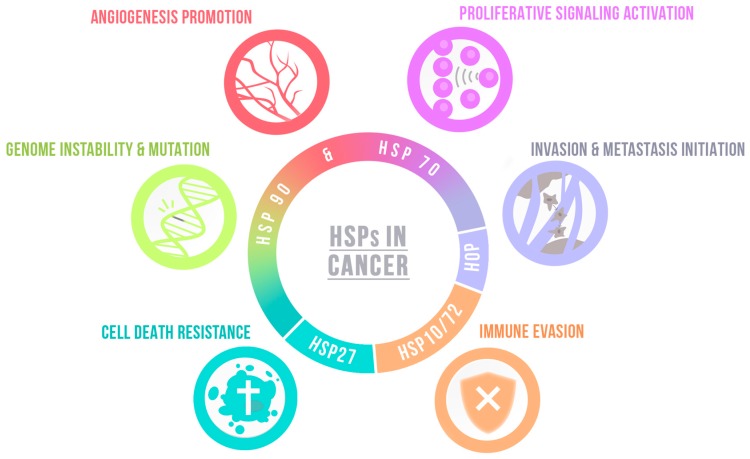
Heat shock proteins (HSPs) modulate several hallmarks of cancer. HSPs work in several mechanisms of proteostasis, features highly relevant in cancer. The figure shows a few of the roles of HSPs in cancer maintenance and are color-coordinated with the hallmark they modulate. HSP70 and HSP90 modulate angiogenesis through regulation of HIF1α and other downstream factors (red). HSP70 and HSP90 preferentially bind to mutated proteins over their wild-type counterparts, stabilizing these molecules and contributing to mutagenesis (green). HSP90 can lead to resistance to cell death by inhibiting the activation of pro-caspase-9, HSP70 inhibits the formation of apoptosomes and pro-apoptotic factors usually released, and HSP27 can inhibit pro-caspase-9 through alternative mechanisms (blue). To evade the host immune system, HSP10 suppresses T-cell activation through T-cell CD3-zeta downregulation, while HSP72 decrease sT-cell proliferation (orange). HSP90 is increased in metastatic tumors and interacts with MMP2, promoting epithelial–mesenchymal transition. HSP70 modulates factors working on cell migration and HSP-organizing protein (HOP), a HSP90/HSP70 co-chaperone, and regulates invasion through MMP2 activation (violet). HSP90 can also modulate self-sufficiency in growth signals, stabilizing several molecules involved in cell proliferation pathways, driving tumor progression and HSP70 through its interaction with ERα, leading to an increased growth in specific tumors (purple).

These findings unveil the key role of HSPs in common traits of cancer biology, and highlight their ability to regulate multiple cancer-related pathways such as cell growth and survival, invasion and migration, and angiogenesis (Figure 1) in distinct types of cancer, including brain tumors. Glioblastoma (GBM) is the most common and aggressive brain tumor, developed from poorly differentiated glial cells characterized by nuclear atypia, cellular polymorphism, and high mitotic activity [46]. GBM usually present abundant cell heterogeneity, diffuse tissue distribution pattern, and extensive dissemination, ultimately resulting in a dismal patient prognostic [47]. 

GBM has a restricted subpopulation of cells within the tumor with stem cell-like characteristics, denominated glioblastoma stem cells (GSCs), able to sustain tumor progression and contribute to therapy resistance [48,49]. These cells have a high capacity for self-renewal, differentiation, invasion, and angiogenesis promotion [50] (reviewed by [51]), besides being responsible for tumor maintenance and relapse after conventional therapy [52]. These cells express classical stem cell markers, such as the CD133 surface marker, and multipotent transcription factor markers, such as Sox2 and Oct4, among others [53]. Interestingly, they inhibit the expression of OCT4/SOX2 or CD133 in human GBM cell line by targeting specifically their promoters, as well as decrease tumor growth both in vitro and in vivo, but fail to eradicate completely the tumor [54].

The limited therapeutic options for GBM are likely a consequence of the malignant properties of GSCs, inevitably resulting in a dreary prognosis for GBM patients. The current standard-of-care involves resection, whether possible, followed by radiation and chemotherapy with temozolomide (TMZ) (Stupp regimen), resulting in a median overall survival (OS) of only 14.6 months [55]. The use of radiotherapy plus concomitant the alkylating agent TMZ increases the patient’s life by 3 years for 16% of patients and by 5 years for about 9.8% of them [56]. However, GBM is able to resist therapeutic interventions by exploiting several molecular strategies [57]. In this review, we discuss new discoveries in light of heat shock proteins and their role in cancer origin and maintenance. In particular, we focus on glioblastomas and GSCs, as well as novel developing therapies against GBM based on HSPs.

## 2. HSPs Function in GBM

The importance of HSPs in cancer, and particularly in GBM, has been previously studied and addressed in different reviews [58,59,60]. While an increasing body of research is focusing on the role of HSP90 and HSP70 in GBM biology, studies investigating the function of HSP60, small HSPs, and HSP100 family are less frequent. Thus, in this section, we highlight recent data (spanning the last 10 years) regarding the function of HSPs and co-chaperones in the biology of GBM (HSPs and their respective function in GBM are listed in Table 1) and their potential as therapeutic targets.

### 2.1. HSP90

Distinct HSP90 clients are involved in important cellular processes such as cell cycle control, protein stability, angiogenesis, cell proliferation, and tumorigenesis. In humans, HSP90 has two isoforms, HSP90α and HSP90β, and is able to bind co-chaperones and co-factors, assembling protein complexes dedicated to assist in maturation of relevant client proteins [9].

HSP90 has been broadly studied as an important factor for GBM cell migration and invasiveness. In particular, inhibition of HSP90 proper functioning has been shown to decrease the migratory capacity in DK-MG and SNB19 GBM cells lines by disrupting the signaling pathways of its clients AKT and MEK. These anti-migratory effects are also associated with cytoskeleton remodeling [61].

Beyond intracellular localization, HSP90 can be found associated with the plasma membrane of cells and in the extracellular environment. Interestingly, extracellular HSP90 (eHSP90) showed to be of significant importance in the aggressiveness of GBM through different mechanisms. eHSP90 interaction with the co-receptor LRP1 (low density lipoprotein receptor-related protein 1) modulates AKT phosphorylation on S897 residue of ephrin A2 (p-EphA2S897), sustaining AKT signaling and lamellipodia formation, and, consequently, increasing GBM cell motility and invasion, which are further enhanced by hypoxia [62]. Other studies explored the importance of the interaction between eHSP90α, EGFR, and the toll-like receptor 4 (TLR-4) in GBM cell mobility. eHSP90α is capable of increasing cytosolic Ca^2+^ and Ca^2+^ oscillations through EGFR in an ATP-dependent manner, and by direct or indirect activation of TLR-4, increasing EGF cellular response, thus facilitating GBM cells migration [63]. Additionally, eHSP90 also modulates GBM invasion and migration through its interaction with heparan sulfate proteoglycans (HSPGs), glycoproteins abundant in the extracellular matrix and cell surface [64].

In addition to GBM cell migration, HSP90 is associated with other processes that influence disease aggressiveness, such as modulation of survival and apoptosis. GBM cell lines submitted to radiotherapy and subsequently confronted with PI-3 kinase and HSP90 inhibitors combined displayed increased cell death and decreased tumor growth in vivo, likely by disrupting AKT signaling, promoting G2/M arrest, and ultimately resulting in apoptosis [65]. Moreover, a novel study correlated HSP90 with the HS-1-associated protein X-1 (HAX-1) in GBM cell lines [66]. HAX-1, a 35 kDa protein, was classically associated with several cellular processes, including B cell signal transduction, cell migration, and apoptosis [67]. HSP90 was identified as an HAX-1 interacting protein, this interaction being important for cardiomyocytes survival [68]. In the context of GBM, HAX-1 and HSP90 interaction appears to facilitate HSP90 association with AKT1, since HAX-1 knockout leads to decreased co-localization and co-immunoprecipitation of both proteins in GBM cell lines (U118 and U87MG), and HSP90 pharmacological inhibition also impairs AKT1 phosphorylation [66]. The disablement of HSP90–AKT1 complex also impacts MDM2 phosphorylation and, consequently, p53 regulation, resulting in cell cycle arrest and ultimately leading to apoptosis in GBM cells [66].

Using an immunohistochemistry approach on a tissue microarray containing different tumor regions, Sartori et al. explored the correlation and prognostic value of HSP90 and EGFR expression in 214 cases of IDH (isocitrate dehydrogenase) wild-type GBM. The study revealed a positive correlation between HSP90 and EGFR expression, especially in the tumor bulk. Unexpectedly, since over-expression of EGFR is commonly associated with more aggressive GBM phenotypes [69], the study found that low co-expression of HSP90 and EGFR was associated with worse prognosis for the disease, indicating an important and unconventional regulatory relationship between these two molecules, although further studies are needed to unravel the molecular mechanisms behind these results [70].

### 2.2. HSP70

HSP70 displays different subcellular localizations, resulting in a plethora of disease-associated roles such as therapy resistance and apoptosis suppression under several stress conditions [15,71,72]. Although previous studies have shown an increase of HSP70 in different cancers, as well as its association with disease maintenance, levels of HSP70 in GBM have only recently been investigated. Analysis of primary and secondary GBM cases of different grades by Thorsteinsdottir et al. showed that not only is the cytosolic form of HSP70 overexpressed in primary GBM, but also its membrane (mHSP70) and secreted form as well [73].

In a rat model of GBM (C6 cells), using pharmacological modulators of HSP70 that control its intracellular levels and its capacity to bind with clients, researchers were able to demonstrate that intracellular HSP70 physically interacts with oxidized, inactive GAPDH in a dose-dependent manner, mainly through its chaperone activity, preventing its aggregation [74]. The aforementioned study emphasizes the protective role of HSP70 in GBM cells undergoing oxidative stress, whereby the activity of GAPDH is critical in such condition, and GAPDH rescue by HSP70 confers resistance and prolongs the survival of GBM cells [74].

Recently, taking advantage of the STED nanoscopy technique, which allows the observation of subcellular structures in intact cells (i.e., super-resolution microscopy), Reindl et al. showed a novel function of mHSP70, participating in tunneling nanotube (TNT) clustering. TNTs mediate unidirectional vesicle and organelle transport through the membranes of different cell types, enabling cell-to-cell connections between different tumor cells. mHSP70, together with the lipid compound globoyltriaosylceramide (GBM3), are important for the architecture of TNT clusters in GBM cells, taking part in this important cellular feature [75].

Glucose-regulated protein of 78 kDa (GRP78/HSP5A) is an endoplasmic reticulum (ER) resident chaperone, a member of the HSP70 family, known to be elevated in several cancers, including GBM [76,77]. In 2018, using in silico modeling and virtual screening methods, researchers found novel possible anticancer compounds based on the inhibition of GRP78 interaction with several ligands. After the screening and the selection of an interaction target, VH1019 and VH1011 were identified as promising compounds, showing antiproliferative effect in different GBM cell lines, with specificity for tumor cells over non-tumors cells [78].

### 2.3. HSP60

Heat-shock protein 60 (HSP60) is another class of proteins induced by stress, expressed in different cellular compartments [79], and commonly considered as a mitochondrial chaperone associated with proteostasis [80]. HSP60 is also expressed in high grade gliomas [81] and, through its interaction with cyclophilin D (CypD), HSP90, and other co-factors, acts by modulating tumor growth and by preventing apoptosis in vivo [82]. HSP60 downregulation leads to EMT, and concomitant production of reactive oxygen species (ROS) in U87MG cells, through a mechanism involving reduction of 4EBP1 phosphorylation and protein translation rates, ultimately affecting cell growth and proliferation [83]. Although these aspects of the role of HSP60 in glioblastoma biology are understood, further studies are still needed to better characterize the role of this molecule in GBM biology.

### 2.4. Small HSPs (sHSPs)

Small HSPs (sHSPs) are a group of HSPs with molecular weight ranging from 15 to 40 kDa, eventually induced by stress, and characterized by the “alpha-crystallin” [84]. In the context of GBM, we highlight the role of two specific proteins: αB crystallin and HSP27. αB crystallin, or HSPB5, is a 175 amino acid protein, highly expressed in vertebrates’ eye lens, heart, muscles, and brain tissue [85]. HSP27 is a chaperone protein highly expressed in several types of cancers, and is considered a potential target for therapy [86]. The stress response properties of αB crystallin in human GBM were identified more than two decades ago in a pioneer study, whereby, due to the structural similarities and the known interaction with HSP27 in other tissues, the authors observed the expression of αB crystallin and HSP27 in U373MG [87]. Furthermore, the same group explored the individual response of both proteins in different GBM cell lines through several types of stress beyond heat shock, such as exposure to arsenite, ethanol, caffeine, and other chemicals. They showed that, although the two proteins produce different effects against thermal and chemical stress induction, both behave in quite similar ways, suggesting that HSPB5 and HSP27 (i.e., sHSPs) belong to a family of HSPs with similar functional roles [87]. Additionally, αB crystallin expression was associated with resistance to cytarabine and β-lapachone treatments, as well as resistance to apoptosis, as induced by cytotoxic cytokines and by increased expression of CD95 and p53 [88].

### 2.5. Co-Chaperones

Co-chaperones are a class of proteins that assist different chaperones in proteostasis maintenance [89]. Co-chaperones are very important in the context of proteome quality control, allowing, along with other co-factors, the assembly of protein complexes and the connection between different chaperones and clients [89,90].

A very common HSP70 co-chaperone, well-studied in the context of GBM, is Bcl-2-associated athanogene domain 3 protein (Bag3). Bag3 is remarkably over-expressed in GBM, and its reduction leads to tumor cell death in vivo and in vitro, especially when combined with other therapeutic strategies [91]. In turn, it was observed that further over-expression of Bag3, beyond its already elevated basal levels in GBM, results in impaired colony formation, likely due to activation of HSF1-driven stress response and accumulation of ubiquitinated clients of Bag3. Such an effect is reverted by disruption of HSP70 binding, and through its WW domain, Bag3 has been implicated in induction of autophagy [92] and resistance to apoptosis [93,94].

DnaJ is also an important co-chaperone for HSP70. Contrary to the expected phenotype of a co-chaperone accompanying the function of HSP70, knockdown of DnaJA1, a DnaJ family member, leads to increased GBM growth and invasiveness, ultimately affecting the OS in animal models [95]. In light of the phenotype displayed by the DnaJA1 co-chaperone, the molecular mechanisms leading to this counterintuitive need to be further investigated. 

Among other co-chaperones, the stress-inducible protein 1 (STI1), also known as HSP-organizing protein (HOP), is another relevant co-chaperone in the context of GBM. HOP mediates the protein complex formed by HSP70 and HSP90 and acts as a molecular “bridge” that allows physical binding of these two chaperones (reviewed in [1]). The proteostasis machinery formed by this complex is highly important for proteome quality control in numerous different cell types, such as pluripotent stem cells (briefly reviewed in [2]), but also in cancer and, in particular, in GBM cells.

HOP expression induces proliferation in different GBM cells types, but not in non-tumor glial cells, through ERK and AKT signaling [96]. Additionally, GBM cells present increased HOP nuclear localization, although the role of this protein in the nucleus of GBM cells remains to be elucidated [97]. In fact, disruption of HSP90–STI1 interaction through an Antp–TPR hybrid peptide leads to ubiquitin-related degradation of several important client proteins, such as p53 and Akt [98]. In 2014, Lopes et al. showed that HOP is increased in GBM tissues when compared to non-tumor tissues and other classes of astrocytomas [99]. Furthermore, the authors showed that the interaction between HOP and cellular prion protein (PrP^C^) is necessary for GBM cell line’s proliferation in vitro and tumor growth in vivo [99].

## 3. HSPs and Glioblastoma Stem-like Cells (GSCs)

Since HSPs are relevant for GBM maintenance and progression, studies are being developed to unveil the roles of HSPs in GSC biology and to explore the therapeutic potential of HSPs.

Filatova et al. demonstrated that necrotic areas of GBM present acidosis and hypoxia, and this niche provides an important scaffold to maintain stemness of GSCs [100]. They identified that HIF modulation in low pH is independent of the classic PDH/VHL (prolyl hydroxylase domain—a component of an E3 ubiquitin ligase complex) path for proteasomal degradation. Indeed, HSP90 maintains HIF1α and 2α expression by increasing their stability [100].

The modulation of HIF by HSP90 leads to increased expression of stem cell markers, as CD133 and HIF target genes as VEGF affecting in vivo tumor growth [100]. It has also been observed that HSP90 expression is located in necrotic areas of biopsies, co-localizing with stem cell markers and HIF [100]. HSP90 loss-of-function, mediated by chemical inhibitors or gene silencing, impairs the expression of HIF during acidosis, with concomitant loss of stemness and tumorigenicity by GSCs [100]. The authors of this study also analyzed gene expression in the glioblastoma cohort of TCGA research network and found an association between HSP90 expression and stem cell markers such as CD133 and nestin, along with HIF target genes. Indeed, HSP90 was correlated with the GSC phenotype, since it is co-expressed with stem cell markers and predominantly observed in hypoxic niches, where it induces HIF transcription factors during acidosis. Moreover, loss-of-function of HSP90 impairs self-renewal and tumorigenicity of GSCs [100]. In detail, the authors demonstrated that GSC maintenance is regulated by the hypoxic and acidotic microenvironment through increased HSP90-dependent expression of HIFs, thus supporting the notion that HSP90 is essential for stemness [100].

In addition, other HSPs play important roles in GSC maintenance. Matsuda et al. demonstrated that heat shock cognate 71 kDa protein (HSC71) binds to nestin and modulates several important functions in GSCs biology [101]. In particular, they demonstrated that: (1) Nestin silencing modulates HSC71 post-translational modifications and nuclear localization; (2) HSC71 knockdown interferes with cell growth, sphere formation, and nestin-dependent cell invasion; and (3) HSC71 and nestin interact to regulate the expression of cyclin D1 [101].

The expression of nestin is restricted to GSC niches, such as perivascular and perinecrotic GBM areas. Reduction of nestin expression impairs sphere formation, in vivo tumor growth, and the expression of stem cell markers such as NANOG, N-cadherin, CD133, and Oct4. HSC71 silencing decreases the expression of nestin and, consequently, several related proteins such as cyclin D1, resulting in impaired cell growth. Moreover, HSC71 depletion negatively modulates sphere formation and invasive capacity of GSCs derived from human GBM cell lines. Together, these findings demonstrate the relevance of HSC71 in modulating stemness through interaction with nestin [101].

HSPs from the HSP70 family are also important for the maintenance of stem cells from different brain tumors. Stem cells derived from medulloblastoma present enhanced expression of HSP70 that, by regulation of its upstream client Akt, activates the NF-κB signaling pathway, which is critical for cell proliferation and survival [102]. HSP70 expression in neuroblastoma models occurs during neuronal differentiation of precursor cells [103] and, different from medulloblastoma, it has no association with the proliferation status [102]. 

The co-chaperone HOP also holds a key role in GSC biology. Through its binding to the PrP^C^, HOP modulates self-renewal and proliferation of GSCs [104]. HOP knockdown impairs GSC proliferation in vitro, as well as tumor growth in vivo. Moreover, a HOP peptide, which mimics PrP^C^ binding site, inhibits GSC proliferation and self-renewal mediated by HOP, and emerges as a potential molecule for GSC-targeted therapy against GBM [104].

HSP47, from the sHSP family, is a collagen-associated chaperone and presents a high expression in gliomas. Enhanced expression of HSP47 in GBM cell lines increases the number of CD44^+^ cells, leading to an enrichment of GSC cells in vitro [105]. HSP47 increased expression also leads to an increase in sphere formation and cell migration in vitro, regulating the expression of extracellular matrix proteins (ECM) and cell surface proteins such as FN1, COL4A2, MMP9 and CD44, LAMC1m, and ITβ1, respectively, through the activation of TGFβ [105]. In vivo, HSP47 modulates invasion, angiogenesis, and tumor formation, also through the activation of the TGFβ signaling pathway, demonstrating an important role of HSP47 in GSC biology and also in GBM maintenance [105].

### Other Chaperones

In addition to HSPs, there are other classes of chaperones that present relevant roles in GSC biology, demonstrating the importance of these molecules for GBM maintenance. For example, histone chaperones, which can modulate epigenetic modifications that lead to a differential pattern of transcription factors expression [2].

FACT is a histone chaperone with essential functions in GSCs, and its expression is associated with high grade of gliomas and poor prognosis, being co-expressed with stem cell markers [106]. FACT levels are enhanced in GSCs compared to bulk tumor cells, its expression decreases during differentiation [106], and FACT’s knockdown leads to a decrease in the expression of transcription factors associated with stemness such as Sox2, Oct4, Olig2, and Nanog [106].

Interestingly, the FACT inhibitor CBL0137 induces GSC differentiation, inhibits the expression of stemness markers, and impairs self-renewal in vitro, while in vivo, CBL0137 decreases GSCs’ tumorigenic capacity and increases survival [106]. Moreover, CBL0137 synergizes with EGFR inhibitors, offering a novel therapeutic option against GBM via GSC targeting [106]. Indeed, FACT inhibition by CBL0137 leads to downregulation of NF-κB signaling and activates p53, which in turn suppresses cell proliferation and increases apoptosis [107]. In vivo, CBL0137 also synergizes with TMZ and may prove effective in non-responsive GBM [107].

DAXX is a histone chaperone that associates with histone 3.3 (H3.3) and forms complexes with PTEN in gliomas, regulating in the expression of oncogenes independent of classic regulation of transcription by the histone enzyme PTRN [108]. DAXX inhibition in PTEN-depleted populations decreases oncogene expression and promotes the expression of tumor suppressors, inhibiting tumor growth in vivo and improving animal survival [108].

Remarkably, the histone chaperone protein ATRX is frequently mutated in GBM and its loss leads to genetic instability [109]. Analysis of genome-wide data in human glioma samples demonstrated an association of ATRX mutation and enhanced single-nucleotide variant (SNV) mutation rate [109]. ATRX knockout models develop genetic instability of SNV mutations, with consequent impairment in survival [109]. Furthermore, tumor response against DNA-damaging agents that cause double-stranded DNA breaks (DSBs) in ATRX-KO mice is improved by impaired non-homologous end joining repair [109]. Together, these data demonstrate that although ATRX loss fosters genetic instability and increases tumor aggressiveness, it also improves the response to chemotherapy based on DSB damage [109].

Nucleophosmin 1 (NPM1) is another histone chaperone with elevated expression in high-grade compared to low-grade gliomas, and its overexpression improves cell survival, while NPM1 depletion leads to apoptosis and increases susceptibility to the chemotherapy agent actinomycin D [110].

Beyond histone chaperones, there are other classes of chaperones with specific functions and locations that have been described as important players in GSC biology. Data from the literature demonstrate that mitochondrial respiration is essential for GSC maintenance, in terms of energy production and survival [111,112]. The chaperone tumor necrosis factor receptor-associated protein 1 (TRAP1) is a mitochondrial paralog of HSP90, which presents a remarkable expression in diverse tumors [113]. The cooperation between TRAP1 and the major mitochondria deacetylase sirtuin-3 (SIRT3) decreases reactive oxygen species (ROS) production and enhances mitochondrial respiration, thus facilitating the stress response and improving cell metabolism, which in turn favors GSC reprogramming [114]. The loss of function of TRAP1 increases differentiation and impairs self-renewal, tumorigenesis, and survival of GSCs through the deregulation of mitochondrial respiration, which is essential for GSC stemness, sphere formation, and tumor growth [115]. Moreover, TRAP1 knockdown in GSCs negatively affects their ability to co-opt for glycolysis (Warburg effect), resulting in decreased tumor growth, cell proliferation and migration, and neurosphere formation, and it also sensitizes GSCs to TMZ [115].

Mitochondrial matrix chaperones are HSPs from different families, and their partners, having specific and relevant roles in mitochondrial proper functioning, include HSP90, 70, and 60 chaperone families [116]. The inhibitor Gamitrinib is an antagonist of TRAP1, characterized by a specific targeting sequence for mitochondria, which inhibits the activity of mitochondrial matrix-associated chaperones. In vitro combination of Gamitrinib with c-Myc inhibitors, such as OTX015, or with c-Myc silencing impairs proliferation of GSCs and promotes apoptosis, while, in vivo, Gamitrinib mediates tumor regression through enhanced cell death and decreased cell proliferation [117].

The Bcl-2-associated athanogene domain 3 (Bag3; see Section 2.5) is associated with the deubiquitinase ubiquitin-specific peptidase 9X-linked (Usp9X), and its downregulation or inhibition by GX15070 leads to apoptosis and decreased proliferation of GSCs in vitro and in vivo by modulating cell death mechanisms [93].

The endoplasmatic reticulum (ER) stress response or unfolded protein response (UPR) is a pro-survival pathway, exploited by GSCs to resist radiotherapy through protein folding by UPR chaperones, such as GRP78 (HSP5A, see Section 2.2) and GRP94 (HSP90 paralog found into ER), which promote autophagy and ER homeostasis [118]. However, recent data from the literature demonstrate that overexpression and overactivation of chaperones from UPR system overload the ER response, leading to radio-induced apoptosis of GSCs, overcoming the radio-resistance of these cells [118]. Moreover, the expression of UPR-related chaperones is inversely correlated with GBM patient survival, supporting the importance of the ER stress response in GSCs radio-resistance [118].

Fatty acid binding protein (FABP) is a family of lipids chaperones that includes FABP7, the chaperone for Omega 3 fatty acid and also a marker of neural stem cells [119]. FABP7 is highly expressed in GSCs compared to cells from the tumor bulk, located at the cytosol and nuclei, and its expression decreases during GSC differentiation [119]. Moreover, FABP7 is co-expressed with Sox2 in gliomas and FABP7^+^/Sox2^+^ cells are found more abundantly in GBMs compared to lower grade astrocytomas, indicating FABP7 as a potential GSC biomarker [119].

Together, these data demonstrate that distinct classes of chaperones play relevant functions in GSC biology and emerge as potential therapeutic targets for anti-GBM therapies based on stem cells properties. 

## 4. HSPs and GBM Therapy

**Figure 2:**
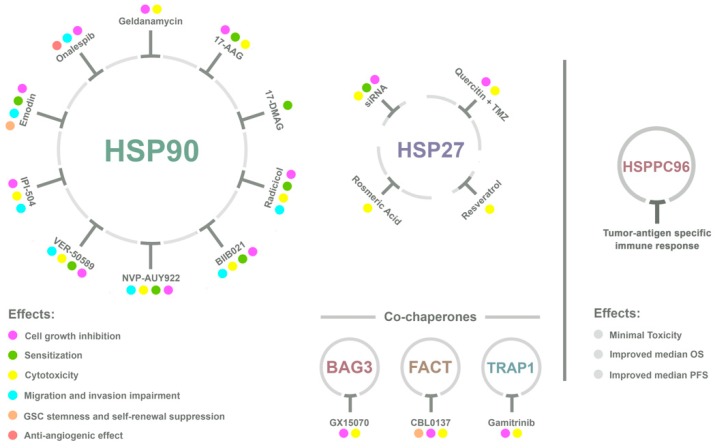
Heat shock proteins (HSPs) targeted for distinct therapeutic approaches anti-GBM. New strategies involve the use of active immunotherapy and HSP-targeting drugs. The most abundant are HSP90 and HSP27 inhibitors, with a few examples of molecules against co-chaperones. The main effects elicited by HSP inhibitors on GBM are cell growth inhibition, radio- and/or chemo-sensitization, cytotoxicity, migration and invasion impairment, glioma stem cells (GSCs) stemness and self-renewal suppression, and anti-angiogenic effect. Vaccines against GBM target the heat shock protein–peptide complex-96 (HSPPC-96) and clinical trials demonstrate that the vaccination causes a tumor antigen-specific immune response on the host, with minimal toxicity and improvement of median overall survival (OS) and median progression-free survival (PFS). Abbreviations: 17-AAG, 17-allylamino-17-435 demethoxygeldanamycin; 17-DMAG, 17-dimethylaminoethylamino-17-demethoxygeldanamycin; IPI-504, Retaspimycin hydrochloride; siRNA, Small-interference RNA; TMZ, temozolomide.

Novel therapeutic strategies against GBM, based on HSPs, include active immunotherapy and target-based drugs (a summary of several HSP-target agents is depicted in Figure 2). Targeted therapies involve the selection of agents based on their mechanism of action. Targets are usually key proteins in dysregulated signaling pathways that promote malignant transformation and tumor growth. The goal of this approach is to improve efficacy and selectivity of tumor treatment by specifically hindering pathogenic mechanisms [120]. Targeted therapy focuses on inhibiting the tumor’s specific genes and proteins that contribute to cancer growth and survival [120]. Due to the observed upregulation of HSPs on brain tumors and their role on glioma proliferation, apoptosis evasion, and metastatic motility, HSPs are being studied as a target for the development of new therapeutic agents against GBM. 

Targeted HSP inhibitors have been evaluated in clinical trials against various cancers [121,122,123], but not for GBM, although preclinical studies have demonstrated efficacy of these agents in GBM models when administered alone or in combination with other therapeutics.

### 4.1. HSP90

HSP90 activity is dependent on adenosine triphosphate (ATP) hydrolysis, thus several inhibitors have been made available that target its N-terminal ATP-binding domain [124]. Most HSP90 inhibitors are ansamycins, purine analogs, or resorcinol derivatives. The ansamycins that inhibit HSP90 activity bind to the protein’s ATP-binding pocket, thereby interfering with the chaperone function [124].

Geldanamycin (GA) is effective in inducing cell-cycle arrest and apoptosis in GBM, but, due to its hepatotoxicity, GA has not made it to clinics [125,126]. Tolerable GA analogs, such as 17-allylamino-17-demethoxygeldanamycin (17-AAG) and 17-dimethylaminoethylamino-17-demethoxygeldanamycin (17-DMAG), show promising results against GBM. 17-AAG sensitizes GBM cells to radiotherapy and to several chemotherapeutics, such as gefitinib (EGFR inhibitor) [127], enzaustarin (protein kinase C inhibitor) [128], LY294002 (PI3K inhibitor) [129], and DNA alkylating agents (TMZ, cisplatin) [130]. The mechanism of action of 17-AAG is similar to its parent compound, i.e., it acts through blockade of HSP90 activity with consequent direct degradation of its client proteins, such as Akt/MAPK/Rb, and abolition of downstream target phosphorylation (e.g., MEK, Cdk4 and 6), independent of GBM mutations in TP53, PTEN, or EGFR. 17-AAG effect is evident in both neural stem cells (NSC) from the Ink4a/ARF tumorigenic model and stem cells isolated from GBM patients, since 17-AAG preferentially targets tumor-initiating cells [131].

Moreover, in GSCs, 17-AAG specifically promotes degradation of HSP90 clients without affecting non-client proteins in the same pathway (PI3K/Akt/mTOR) [131]. Through HSP90 inhibition, 17-AAG leads to decreased tumorigenic capacity and tumor growth, and increased cell death of GSCs, ultimately resulting in improved OS in vivo [131]. Interestingly, neuroblastoma-derived stem cells are also more susceptible to the 17-AAG HSP90 inhibitor than differentiated cells, presenting decreased cell proliferation [132].

In 2010, Ohba et al. investigated the inhibition of HSP90 as a possible approach to increase the cytotoxicity of chemotherapeutic agents in GBM cells [133]. The group demonstrated that pharmacological inhibition of HSP90 by 17-AAG leads to increased sensitization of these cells to DNA-crosslinking agents such as cisplatin and 1,3-bis (2-chloroethyl) -1-nitrosourea (BCNU) in a dose-dependent manner, by suppressing the cell cycle regulators proteins CDC2 and CDC25C, generating a G2/M arrest, and increasing apoptosis, while suppressing Akt and survivin [133]. Moving forward to clinical trials in cancer, although 17-AAG showed good tolerability in phase I trials [134], the drug showed a lack of clinical activity in a phase II trial against metastatic pancreatic cancer [135].

In U251 glioma cells, 17-DMAG decreased ErbB2, c-Raf, and Akt protein levels, in addition to sensitizing cells to radiation treatment. In U251 and T98G cells, 17-DMAG enhances the radiosensitizing effect of TMZ [136]. Similar to 17-AAG, no clinical trials were performed with 17-DMAG in GBM. Moreover, Gaspar et al. 2009 showed acquired resistance to 17-AAG and cross-resistance with 17-DMAG in four human GBM cell lines [137]. HSP90 inhibitors such as Radicicol, BIIB021, NVP-AUY922, and VER-50589, which lack a quinone moiety, did not demonstrate cross-resistance in 17-AAG-resistant GBM cell lines [137]. Treatment with the aforementioned inhibitors as single agents demonstrated cytotoxicity, decrease of tumor cell motility, radiosensitization, antiproliferative effects, and degradation of HSP90 client proteins such as EGFRvIII [138,139,140,141,142].

In GBM cell lines and patient-derived spheres, HSP90α and HSP90β are both highly expressed in CD133 positive as compared to negative cells, and their expression decreases during differentiation. Conversely, basal levels of HSP90α and HSP90β are low in NSCs and increase during differentiation. Indeed, an increase in the levels of CD133 correlates with HSP90α and HSP90β expression, as well as with sphere formation capacity [143].

The HSP90 inhibitor Retaspimycin hydrochloride (IPI-504) provokes toxicity to GSCs only, while, at comparable dosage of inhibitor, NSC continues to grow normally. In GSCs, IPI-504 leads to a decrease in cell proliferation, disrupts cell cycle, and promotes apoptosis independent of conventional treatment, since its effect was observed in both TMZ sensitive and resistant cell lines [143]. Moreover, HSP90 inhibition by IPI-504 impairs GSC migration and invasion and might affect angiogenesis, since it decreases the levels of HSP90 clients such as EGFR, ultimately regulating VEGF secretion [143]. However, in vivo IPI-504 treatment presented modest effect, slowing the growth of GSC-derived tumors and leading to regression only in a few cases. Indeed, although levels of HSP90 and its clients were decreased, the lack of efficacy of IPI-504 could be explained by HSP70 upregulation, likely from a compensatory mechanism [143].

Kim et al. observed that 1,3,8-trihydroxy-6-methylanthraquinone (emodin), a component of *Rheum palmatum* root, suppresses stemness of GSCs by leading to proteasomal degradation of EGFR, following impairment of its association with HSP90 [144]. Emodin is capable of interfering with the expression of Notch intracellular domain, total β-catenin, and phosphorylation of STAT3, all of which are relevant for stemness maintenance, self-renewal, and invasiveness. Moreover, emodin sensitizes GSCs to ionizing radiation promoting apoptosis, thus presenting as a potential adjuvant therapy for GBM, tailored to GSCs by targeting the expression and activation of HSP90 clients [144].

Onalespib, a second-generation HSP90 inhibitor showed longer duration of inhibition and an adequate toxicity profile in phase I studies in patients with non-CNS solid tumors [145,146]. Recently, onalespib was tested in combination with TMZ in GBM zebrafish and mouse xenografts, and led to extended survival in these animal models [147]. Moreover, inhibition of HSP90 by onalespib disrupted cell signaling of several HSP90 client proteins and decreased proliferation, migration, and angiogenesis of glioma cells lines and patient-derived glioma-initiating cells [147]. In addition, onalespib crosses the blood–brain barrier, an important ability required for GBM chemotherapeutics.

### 4.2. HSP70 and HSP27

Targeted anti-HSP27 strategies have shown limited efficacy due to the dynamic structure of the protein and the scarcity of direct ligands [148]. Moreover, since HSP27 activity is independent of ATP hydrolysis, the strategy of designing specific nucleoside binding site inhibitors is not a possibility, as it is for HSP90 inhibitors. The strategies currently in use for disrupting HSP27 expression and function are gene silencing with small interfering RNA (siRNA) and antisense oligonucleotides. A few small molecule inhibitors that specifically target HSP27 are still in early development [130]. Attenuation of HSP27 expression by siRNA sensitizes GBM cells to irradiation [149] and decreases GBM cell proliferation and viability, while also sensitizing cells to TMZ treatment [150]. Furthermore, HSP90 inhibitors increase HSP27 expression, while concurrent treatment with HSP27 siRNA enhances cytotoxicity of the HSP90 inhibitor [151].

Quercetin, a bioactive flavonoid, causes growth inhibition and cell death in a variety of cancer cells, including human GBM cells [149,151]. TMZ combined with quercetin induces apoptosis via an increase in caspase-3 activity in GBM cells [152]. TMZ alone increases phosphorylation of HSP27 in U251 and U87 GBM cells, while co-treatment of TMZ and quercetin or HSP27 siRNA attenuates HSP27 phosphorylation and inhibits HSP27 expression [152]. Barbarisi et al. synthesized a nanocarrier of quercetin combined with TMZ targeting the CD44 receptor on GBM cells [153]. This nanocarrier increased the internalization of quercetin and TMZ, enhancing the cytotoxicity while reducing the production of IL-8, IL-6, and VEGF by GBM cells.

Rosmarinic acid (RA) is a natural antioxidant that has been shown to possess antitumoral effects. In human GBM cells, RA alone decreased HSP27 protein levels and induced apoptosis. When combined with HSP27 siRNA, RA suppressed HSP27 expression by 90.5% and demonstrated a 58% increase in caspase-3 activity [154]. Resveratrol showed a similar effect as RA on human GBM cells, decreasing HSP27 protein levels and inducing apoptosis, with these effects being potentiated by combined treatment with HSP27 siRNA [155].

Although these natural antioxidants show promising efficacy against GBM, an in vivo study demonstrated that treatment with 50 mg/kg of quercetin for 15 days on a glioma implantation rat model highly increased tumor volume [156]. The authors suggest that this effect may be due to the low concentration of 0.53 µM of quercetin found in the brain of the animals after 15 days of treatment. In vitro studies use much higher concentrations of quercetin, with toxic concentrations for several cancers being in the range of 20 to 100 µM. In fact, to date, there are no positive results on the use of quercetin against cancer in clinical trials. As such, a clear-cut effect of these natural compounds should be demonstrated in animal models before their use on humans.

Despite the studies presented here, and the ongoing research on co-chaperones, additional information about the involvement of these specific proteins in GBM would be crucial to better understand the biology of this deadly disease. The studies demonstrating HSP-targeted inhibition and acquired resistance of GBM cells against these agents suggest that an appropriate strategy would be to use inhibitors that target more than one HSP, co-chaperones, and their clients. HSP inhibitors have demonstrated favorable results in phase I/II clinical trials for non-CNS tumors. Along with the use of standard GBM chemo- and radiotherapy, HSP inhibitors could potentially improve the response of GBM patients to therapy. To date, however, no clinical trials with HSP or co-chaperone-targeted inhibitors have been performed against GBM.

### 4.3. HSP Vaccines Against GBM

Active immunotherapy educates näive immune cells of the host against specific antigens. An example are vaccines which elicit a specific immune response through injection of foreign antigens. The antigens are taken up by resident antigen-presenting cells (APCs) and are presented to lymphocytes [157]. In the case of HSP vaccines against GBM, HSP–peptide complexes are isolated and purified from resected GBM, and are subsequently reinfused into the patient in order to induce an immune response against those tumor antigens [158].

HSP vaccines are composed of HSPs and tumor–antigenic peptide complexes (HSPPCs), and this vaccine can stimulate both the innate and adaptive immune response [159,160]. Individual HSPs or autologous tumor–antigenic peptides alone lack the ability to elicit an immune response; only HSPPCs can be endocytosed by APCs and activate CD4^+^ and CD8^+^ T cells, triggering an adaptive immune response against autologous tumor peptides [159,160].

HSPPCs can also interact with several APC surface receptors and induce downstream activation of the NF-κB pathway [161]. Particularly in macrophages, HSPPCs induce the secretion of proinflammatory cytokines such as IL-12, IL-1β, tumor necrosis factor α (TNFα), and granulocyte colony-stimulating factor (GM-CSF). IL-12 can activate cytotoxic activity on lymphocytes and natural killer (NK) cells. Moreover, HSPPCs are capable of increasing the production and secretion of nitric oxide on macrophages and dendritic cells [161]. HSPPCs can also induce maturation of dendritic cells, further potentiating a proinflammatory response [162].

Another advantage of HSP vaccination is that it targets more than one tumor antigen. Since HSPs can form complexes with several different tumor peptides, they can present various types of antigenic proteins to APCs [158]. This is important to try to circumvent the intratumoral heterogeneity of GBMs. A patient-specific polyvalent HSPPC vaccine may be more advantageous compared with a single target vaccine. Another benefit of HSPPC vaccines is their ability to increase cytotoxic effects through cell types other than cytotoxic T-lymphocytes (CTLs) [158].

The most used HSPPC for vaccines is the heat shock protein–peptide complex-96 (HSPPC-96), which is composed by autologous antigenic peptides chaperoned by HSP glycoprotein 96. An early phase I clinical trial (NCT00293423) has shown HSPPC-96 to be safe with minimal toxicity, composed of mild injection site erythema and/or induration [163]. Moreover, the HSPPC-96 vaccine elicited a tumor antigen-specific peripheral immune response in the tumor and peripheral blood in 11 of 12 patients with recurrent GBM after vaccine administration. In this study, the vaccination schedule was comprised of 25 µg HSPPC-96 every 2 weeks for a total of 4 vaccinations, or 25 µg HSPPC-96 weekly, totaling 4 vaccinations. After this first round of vaccination, patients were administered a biweekly dosing. Seven patients undertook subsequent tumor biopsies after the vaccine doses, which showed the presence of IFN-γ positive NK and T immune effector cells in the tumor site. Moreover, the vaccine was associated with a median OS of 47 weeks for the immune responders, compared with 16 weeks for one non-responder [163].

An open-label, single-arm, phase II clinical trial (NCT00293423) with the HSPPC-96 vaccine enrolled 41 patients that underwent gross total resection of recurrent GBM and received a median of 6 vaccinations [164]. Median OS was 42.6 weeks. The authors also observed that patients with lymphocyte counts below the cohort median had decreased OS. Additionally, only a single grade 3 adverse event related to the vaccine was observed.

A following open-label single-arm, phase I clinical trial (NCT02122822) enrolled 20 patients with newly diagnosed GBM. All patients underwent tumor resection and received the Stupp regimen [165]. Vaccination was administered concurrently with the beginning of TMZ chemotherapy and was composed of 25 µg doses every week for 6 weeks. Tumor-specific immune response (TSIR) increased by 2.3-fold after vaccination. In a similar way to what was observed for the first phase I trial, median OS for patients with high TSIR was 40.5 months compared with 14.6 months for patients with low TSIR. Additionally, median progression-free survival (PFS) was longer for patients with high TSIR (12.3 months) compared with patients with low TSIR (9.0 months).

A recently completed phase II trial (NCT00905060) observed a median OS of 23.8 months for patients that received the HSPPC-96 vaccine after surgical resection, followed by radiation and chemotherapy [166]. Bloch et al. 2017 also evaluated PD-L1 expression in peripheral myeloid cells as a predictor of survival. They found that patients with high PD-L1 expression had a median OS of 18.0 months, compared with 44.7 months for low PD-L1 expression [166].

Another phase II study (NCT01814813), however, failed to display a survival benefit for vaccination with HSPPC-96 alone or in combination with the monoclonal antibody bevacizumab compared to bevacizumab alone. Another phase II trial is currently in the phase of recruiting patients and it is analyzing the potential additive effects of the HSPPC-96 vaccine, along with the administration of the anti-PD1 antibody pembrolizumab and with standard radiation therapy and TMZ. This study shows promise due to the results observed on patients with low PD-L1 expression on peripheral myeloid cells in the NCT00905060 phase II trial. The major possible drawback, however, for the HSPPC-96 vaccine against GBM is acquiring enough quantities of purified HSP-bound peptides for vaccine formulations.

## 5. Conclusions and Perspectives

Post-translational mechanisms and proteostasis are increasingly being considered as essential study subjects for the understanding of tumor cell biology. Given the characteristics of GBM, with its increased aggressiveness and poor prognosis for the patients, all aspects that may be leading these cells to a high degree of survival, even in the face of different treatments, should be considered. As we highlight in this review, different proteins from the HSP family play a key role in GBM cell migration, invasion, and survival. Although many studies address the function of HSP90 and HSP70 family-specific proteins, more studies that encompass other HSP proteins, such as HSP60 and HSP100, need to be performed to better understand the function of these molecules in the context of the disease. Despite the lack of clinical studies, the use of HSP inhibitors combined with conventional GBM treatments, such as TMZ or radiotherapy, appears to be a promising therapeutic approach based on positive results observed in vitro, with interesting perspectives for targeting stem-like cells. Nevertheless, further studies that consider this combinatorial approach should be fulfilled in different systems before being used in clinical trials.

**Table 1:** Role of HSPs and co-chaperones in glioblastoma (GBM) biology. GSCs, glioblastoma stem-like cells; EMT, epithelial–mesenchymal transition.

Hallmark		Chaperone	Function
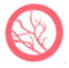	Angiogenesis promotion	HSP90	Modulates HIF, consequently VEGF [100]
		HSP47	Promotes angiogenesis activating TGFβ signaling pathway [105]
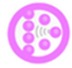	Proliferative signaling activation	HSC71	Modulates cyclin D1 expression in GSCs [101]
		HSP60	Increases cell growth, regulates ROS production and EMT [83]
		DnaJ	Increases cell growth in vivo [95]
		HOP	Promotes proliferation in vitro and GBM growth in vivo [99]
			Modulates self-renewal and proliferation of GSCs [104]
		FACT	FACT inhibition supresses self-renewal and sternness of GSCs [106,107]
		DAXX	DAXX inhibition decreases tumor growth and improves survival [108]
		TRAP1	TRAP1 depletion decreases sphere formation of GSCs and tumor growth [114,115]
		Bag3	Bag3 silencing impairs cell proliferation in vitro and in vivo [93]
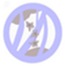	Invasion and metastasis initiation	HSP90	Promotes cells migration and invasiveness [61,62,63,64]
		HSC71	HSC71 knockdown impairs nestin-dependent cell invasion [101]
		HSP47	Promotes cell migration and ECM proteins expression in GSCs [105]
		DnaJ	Increase tumor invasiveness [95]
		TRAP1	TRAP1 knockdown decreases migration by disturbing the Warburg effect [115]
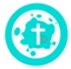	Cell death resistance	HSP90	Modulates apoptosis resistance in vivo and in vitro [65,68]
		HSP70	Regulates oxidative stress protection and cell survival [74]
		HSPB5	Modulates apoptosis resistance [88]
		FACT	FACT inhibiticm increases apoptosis [106,107]
		NPM1	NPM1 depletion increases apoptosis and susceptibility to chemotherapy [110]
		TRAP1	TRAP1 loss impairs GSCs survival [114,115]
		Bag3	Regulates autophagy, promotes apoptosis resistance and survival in vivo [91,92,93,94]
		HSP5A	HSP5A overactivation induces radio-induced apoptosis of GSCs [118]
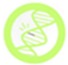	Genome instability & mutation	ATRX	ATRX loss leads to genetic instability and increases tumor aggressiveness [109]
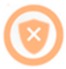	Immune evasion	HSPs contribute to antigen presentation—See Section 4.3.
